# Ensuring protocol compliance and data transparency in clinical trials using Blockchain smart contracts

**DOI:** 10.1186/s12874-020-01109-5

**Published:** 2020-09-07

**Authors:** Ilhaam A. Omar, Raja Jayaraman, Khaled Salah, Mecit Can Emre Simsekler, Ibrar Yaqoob, Samer Ellahham

**Affiliations:** 1 Department of Industrial & Systems Engineering, Khalifa University, Abu Dhabi, 127788 United Arab Emirates; 2 Department of Electrical Engineering and Computer Science, Khalifa University, Abu Dhabi, 127788 United Arab Emirates; 3 Heart & Vascular Institute, Cleveland Clinic Abu Dhabi, Abu Dhabi, United Arab Emirates

**Keywords:** Blockchain, Clinical trials, Healthcare, Ethereum, Smart contracts, IPFS

## Abstract

**Background:**

Clinical Trials (CTs) help in testing and validating the safety and efficacy of newly discovered drugs on specific patient population cohorts. However, these trials usually experience many challenges, such as extensive time frames, high financial cost, regulatory and administrative barriers, and insufficient workforce. In addition, CTs face several data management challenges pertaining to protocol compliance, patient enrollment, transparency, traceability, data integrity, and selective reporting. Blockchain can potentially address such challenges because of its intrinsic features and properties. Although existing literature broadly discusses the applicability of blockchain-based solutions for CTs, only a few studies present their working proof-of-concept.

**Methods:**

We propose a blockchain-based framework for CT data management, using Ethereum smart contracts, which employs IPFS as the file storage system to automate processes and information exchange among CT stakeholders. CT documents stored in the IPFS are difficult to tamper with as they are given unique cryptographic hashes. We present algorithms that capture various stages of CT data management. We develop the Ethereum smart contract using Remix IDE that is validated under different scenarios.

**Results:**

The proposed framework results are advantageous to all stakeholders ensuring transparency, data integrity, and protocol compliance. Although the proposed solution is tested on the Ethereum blockchain platform, it can be deployed in private blockchain networks using their native smart contract technologies. We make our smart contract code publicly available on Github.

**Conclusions:**

We conclude that the proposed framework can be highly effective in ensuring that the trial abides by the protocol and the functions are executed only by the stakeholders who are given permission. It also assures data integrity and promotes transparency and traceability of information among stakeholders.

## Background

The rapid advancements in blockchain and its prominent features that enforce transparency, trust, and data integrity have expanded its scope beyond the finance sector [1, 2]. There are mainly two types of blockchain networks, namely, public and private. The former is an open-source network that allows anyone to participate in the network; whereas, the latter places a restriction on who is allowed to enter and participate in the network [3]. The unprecedented potentials of blockchain have enabled its wide adoption in various industries and domains, e.g., supply chain, engineering maintenance, IoT [2, 4], AI [5], deepfake videos [6], and the healthcare sector [7]. For instance, IBM has shown that 56% of healthcare administrators plan on implementing blockchain-based solutions by 2020 [8]. Blockchain has significantly revolutionized the way Clinical Trial (CT) data is stored, transmitted, and managed. Modern CTs face several challenges related to huge volume and variety of data generated during multiple phases and multi-year studies. These include privacy concerns [9, 10], increased expenses [11, 12], reproducibility of results [13], patient enrollment/recruitment [14], data integrity [15], protocol compliance, and data sharing [16]. To tackle these obstacles, researchers are compelled to move away from legacy clinical database management platforms, such as Oracle Clinical software and SigmaSoft’s DMSYS Software [17]. Thus, the CT industry has been keen on exploring different blockchain platforms, such as Hyperledger [18] and Ethereum [19] to provide feasible solutions to combat data management issues.

Blockchain enhances CT research by empowering the research community with a secure network of sharing data. Typically, blockchain is a distributed ledger with all valid transactions stored as a chain of blocks. These transactions cannot be stolen, hacked or infringed since the data is stored over a decentralized network where each node has a copy of the database [8]. The nature of the network, verification requirements through cryptographic techniques and timestamped records ensure that data is immutable and auditable [20, 21]. Hence, it is a safe platform for storing sensitive information including electronic healthcare records, clinician notes, e-prescribing, analysis results, and protocol of CT. Ethereum is one of the blockchain platforms which runs smart contracts. It allows the creation of smart contracts using Solidity language. It is a high-level language whereby one can develop contracts using various components, such as variables, addresses, setters, getters, events, and modifiers. Such contracts enable users to program different functionalities and build various applications and services. The contracts act as smart software agents to automatically administer certain transactions when preassigned conditions are met in a blockchain network [22, 23], which make them suitable for CTs as they eliminate the need for third-party interference. In the Ethereum smart contract system, initially, each actor needs to be registered through a unique Ethereum address (EA) that is stored in the Ethereum Wallet. The wallet requires a private key, specific to the individual actor that enables access to the Ethereum address stored in the wallet, which is also the public key. The public key from the wallet authorizes access to actors responsible to perform certain activities. Any unauthorized actor cannot access the smart contracts due to a mismatch between private-public key pairs stored in the wallet. In this way, the Ethereum user authentication is handled or managed. Storing vast amounts of data on the blockchain is expensive and energy-draining [24]. For data storage, decentralized mechanisms, such as InterPlanetary File System (IPFS) [24] and FileCoin [25] are currently being used along with blockchain systems whereby stored data is traceable and immutable.

The convergence of blockchain and CTs is still in its infancy and as such very limited literature is available on this subject. For example, Choudhury et al. [26] have proposed a framework that helps to manage and monitor data in multi-site CTs. In [27], the authors have developed a system named “BlockTrial”, which facilitates users to execute trials-related smart contracts on the Ethereum network. Such contracts grant researchers access to their data and enable them to submit queries for data stored on off-chains. The study conducted in [28] implements a blockchain-based solution that enables patients and stakeholders to manage consent information in a manner that is transparent, immutable, traceable, and secure. Another study [29] has introduced a framework, which helps to deal with the dynamic nature of consent management. The authors in [30] proposed a blockchain-based framework that aims to enforce Institutional Review Board (IRB) regulations (i.e., subject recruitment, informed consent management, secondary data sharing, monitoring risks, and generating automated assessments for continuous review) on data collection. In summary, existing literature shows that researchers have investigated various blockchain platforms for CTs to evaluate their capabilities and limitations. However, only a few studies including [31] have tailored their work towards creating consensus algorithms and possible architectures suitable to meet the needs of CTs. Several other studies have highlighted the significant problems in CTs that blockchain can potentially solve [16, 20]. Such problems include patient privacy and enrollment, regulatory approval, and data integrity and reproducibility. Leveraging blockchain technology for managing CT data can help to stimulate innovations and bring major improvements in existing CT data management systems. In this paper, we propose a blockchain-based solution to address the challenges associated with CTs data management. Specifically, the paper provides a proof-of-concept design that addresses the data management, protocol compliance, data integrity, and transparency-related challenges in CTs. This paper is significantly extended from preliminary results presented in [32]. The main contributions are summarized below:

- We provide an overview of how blockchain can enable effective CT data management processes.
- We propose a blockchain-based framework detailing the relationship and interaction between various stakeholders in a CT process using a smart contract and IPFS.
- We present algorithms used to define the working principles of the proposed blockchain approach in a CT data management system.
- We test various scenarios of the overall system functionalities to validate the practicality of the proposed solution.
- We present the full implementation details and make the smart contract code publicly available on Github.

## Methods

The proposed solution is based on the Ethereum blockchain platform to enable effective CT data management.

### System overview and design

**Fig. 1:**
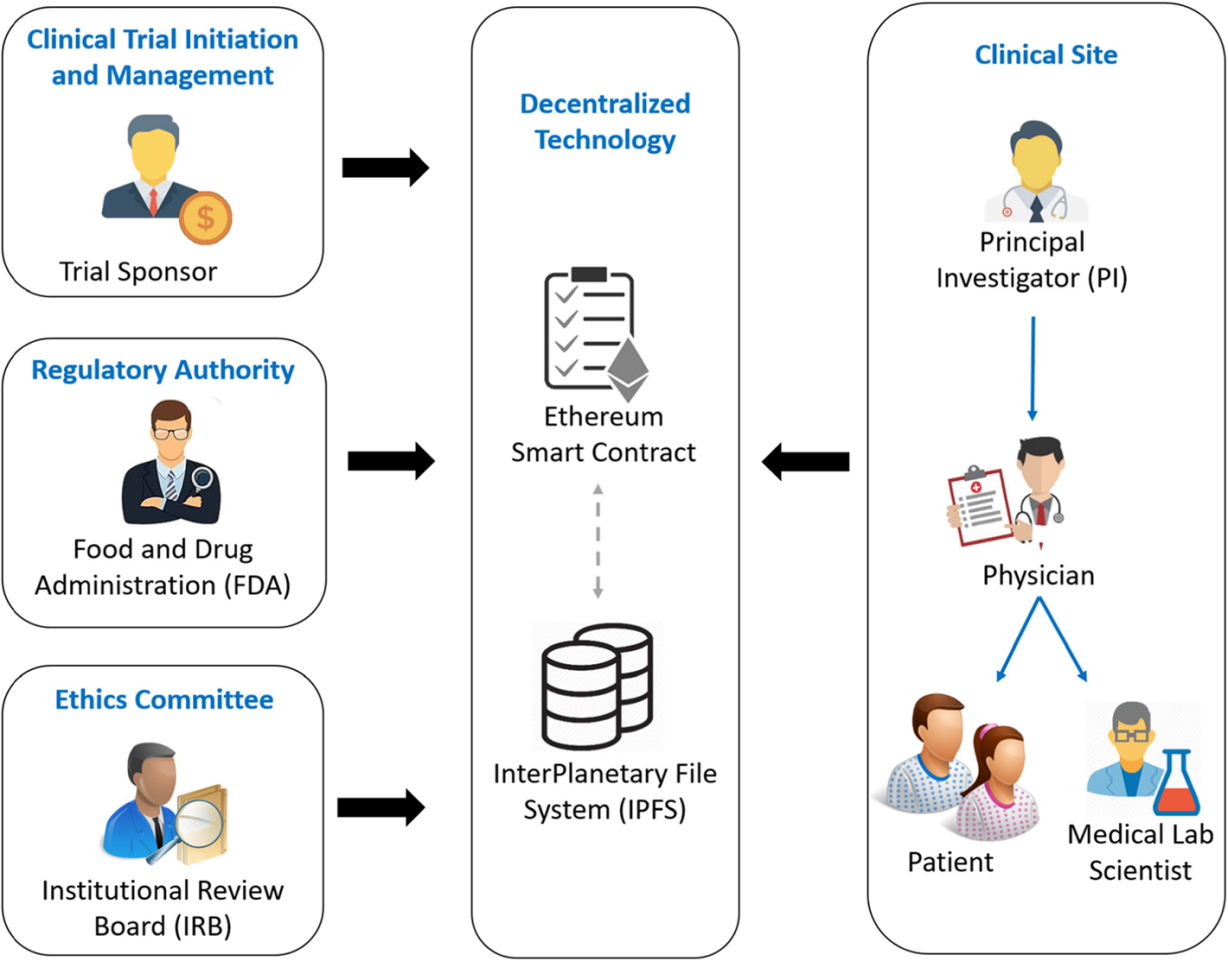
An overview of a CT process using smart contract and IPFS

**Fig. 2:**
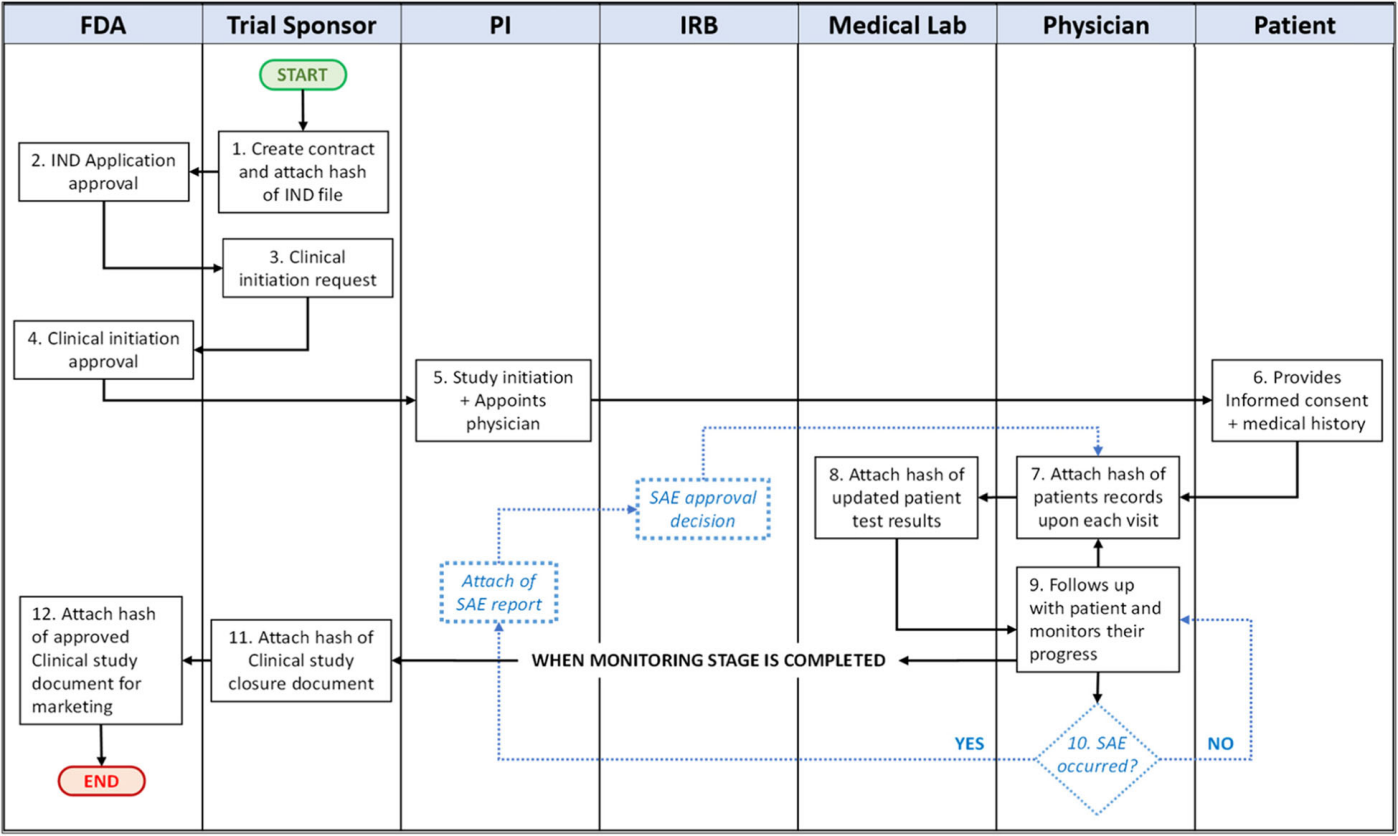
Process flow of the proposed system

Figure 1 presents a system overview of the CT process. This process is explained in twelve steps as shown in Fig. 2. The stakeholders in the proposed system can be divided into four groups. The clinical trial initiation and management are carried out by the trial sponsor; whereas, the regulatory authority and ethics committee are handled by the Food and Drug Administration (FDA) and IRB. Other stakeholders involved in the clinical site are principal investigator (PI), physician, and medical lab scientist. In our system design, a physician is responsible to contact with patients and medical lab scientists simultaneously to monitor and track the patient’s progress. The proposed system architecture places the Ethereum smart contract at the center of the model where stakeholders can actively interact with the contract and one another. By doing so, it aims to decentralize data management in CT by potentially eliminating the role of Contract Research Organization (CRO). In traditional systems, CT data is monitored and controlled by the CRO. Thus, the responsibilities of the CRO would be ideally transformed into a smart contract for automatic execution eliminating any interventions. Moreover, smart contract features can constantly track and monitor the transactions and when they occur through the execution of functions and triggering events that would act as alerts notifying members. Moreover, protecting the identity of patients would be easier as each patient would have an encrypted address mapped to their identity where their information can be accessed only in two ways. Either the patients agree to provide their private keys to the designated trial site or the trusted appointed physician creates and holds the patients’ keys throughout the CT process to monitor their response with regards to the drug treatment. In our case, we chose the latter alternative as the physician would be able to understand the progress of the patient better and explain it to the respective patient during their appointed follow-up visit. This is beneficial as the physician is more likely to avoid misinterpreting the results compared to the patient.

As shown in Fig. 1, the framework incorporates IPFS technology to store a collection of hashed files that could be retrieved anytime when incorporated within the blockchain. Files stored on the IPFS network are given a unique cryptographic hash which is later used to track the corresponding file. This makes IPFS an ideal place to store data as files are traceable, immutable and timestamped via blockchain. Examples of documents that could be stored in the IPFS include but are not limited to Investigational New Drug (IND) application, protocol, Standard Operating Procedure (SOP), Case Report Forms (CRFs), PI’s resume, patients consent and medical history, Serious Adverse Event (SAE), lab tests, financial and safety reports and lastly, clinical study closure report. IPFS permits users to make valid changes during the entire CT life cycle. For example, in a certain case, where changes need to be made in the document after uploading it to the IPFS, a user can reupload the modified version of the document on the IPFS against a new hash value that can be subsequently stored on the blockchain. In this way, a legitimate change or any protocol amendment can be accommodated after uploading the document to the IPFS.

Furthermore, Fig. 2 shows the flow diagram of our proposed system, which presents activities occurring in sequence or parallel. It should be noted that patients do not directly interact with the smart contract.

### Entity relationship diagram

**Fig. 3:**
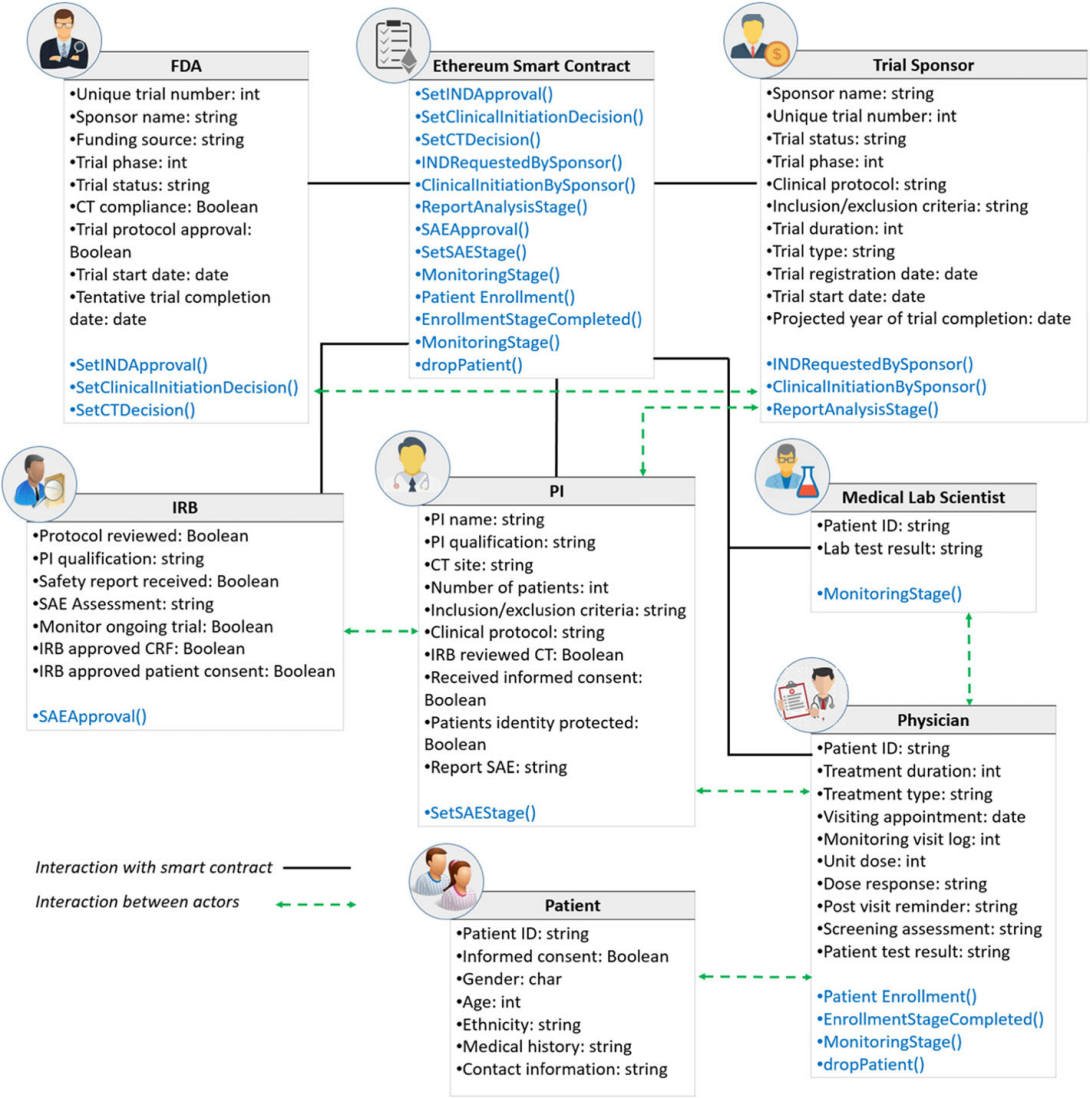
Entity-relationship between different stakeholders and smart contracts

Figure 3 illustrates the attributes of smart contracts along with their functions. In addition, it shows the relationship between different stakeholders and smart contracts. These relations and metadata are vital in implementing smart contracts.

Furthermore, the relationship between any entity and the contract is one-to-one as the stakeholders were assumed to be single entities (i.e., FDA, IRB, trial sponsor, PI, physician, and medical lab scientist). Also, the CT was assumed to be conducted across a single clinical site with multiple patients. As a result, only the relationship between the physician and patients is one-to-many.

### Sequence diagram

**Fig. 4:**
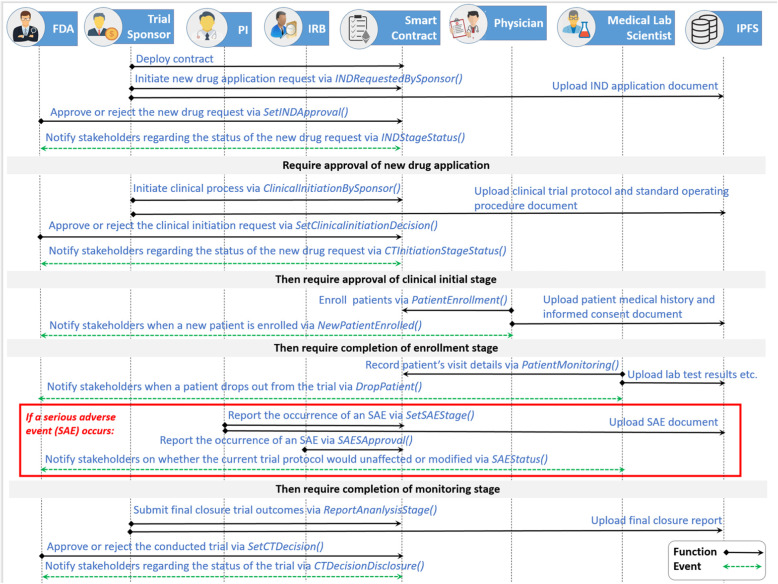
Sequential illustration of function calls and events in a blockchain-based CT data management system

A sequence diagram shows the interactions between different stakeholders while simultaneously showing various events that are triggered in the sequence of functions that are called within the smart contract. Each participant in the network holds an Ethereum Address (EA) that enables them to interact with each other by calling functions within the smart contract. Figure 4 illustrates the sequence flow between different stakeholders from the IND application to the final step of reporting the results obtained by the CT study.

Initially, the CT trial sponsor is granted permission to apply for an IND approval by executing the function *INDRequestedBySponsor()* where this function would include attributes, such as IPFS IND application, trial phase intended, expected trial duration, etc. The IPFS IND application is nothing but the hashed IND application document that is uploaded into the system by the trial sponsor where the original file could be retrieved through IPFS. When the trial sponsor confirms that the application is completed, then the FDA calls the function *SetINDApproval()* where the decision of approval or rejection is made. Upon receiving the decision, the event *INDStageStatus()* is triggered which notifies the trial sponsor and IRB whether the application is approved or rejected. In case of application approval, then the trial sponsor calls the function *ClinicalInitiationBySponsor()* where accordingly inputs, such as IPFS clinical protocol and IPFS SOP, are uploaded into the smart contract. Once this step is completed by the trial sponsor, only then the FDA is allowed to interact with the *SetClinicalInitiationDecision()* function and dictate whether the protocol has been approved or rejected. This invokes the event *CTInitiationStageStatus()* to notify the trial sponsor, PI and IRB that the trial will now be ready to commence recruiting patients according to the inclusion-exclusion criteria.

Then the physician assigned by PI calls the function *PatientEnrollment()*to upload patient’s EA, hashed medical history and informed consent etc. Furthermore, an event *NewPatientEnrolled()* is triggered whenever a new patient is registered. It should be noted that the patients do not interact with the smart contract. Hence, they are passive participants and their communication with the physician is via off-chain methods, such as face to face appointments. Furthermore, the patients would be distinguished by their EA, thereby securing their personal identification information. The trial commences only when the enrollment is complete. Then the physician and medical lab scientist call the function *MonitorStage()*to record the patient’s weekly or monthly visits, the patient’s hashed lab results etc. where this process continues until the trial period ends. When a patient drops out from the trial, then the physician triggers the *PatientDroppedOut()* event to notify all members in the network that this particular patient is no longer participating and no further monitoring is needed. At the end of the trial, statistical computation and a final closure report are required to be uploaded by the trial sponsor by calling the *ReportAnalysisStage()* function. These report generations and statistical analyses could be done using decentralized applications (DApps) where these are applications designed to run on a peer to peer network, such as blockchain. DApps have recently gained popularity due to distributed ledger technologies, such as Ethereum.

Lastly, the final decision comes from the FDA where this decision is made by calling the *SetCTDecision()* function. This decision is then announced when the event *CTDecisionDisclosure()* is triggered. This publicly notifies the decision made by the FDA to all the active participants in the CT blockchain network. However, under the circumstance where an SAE occurs during the trial monitoring process, then the PI calls the function *SetSAEStage()* where the IPFS SAE report is uploaded. Then the IRB calls the *SAEApproval()* function to make a decision on the SAE that has occurred. The *SAEStatus()* event is triggered to broadcast the decision taken by the IRB regarding the continuation of the current trial protocol.

### Implementation

**Fig. 5:**
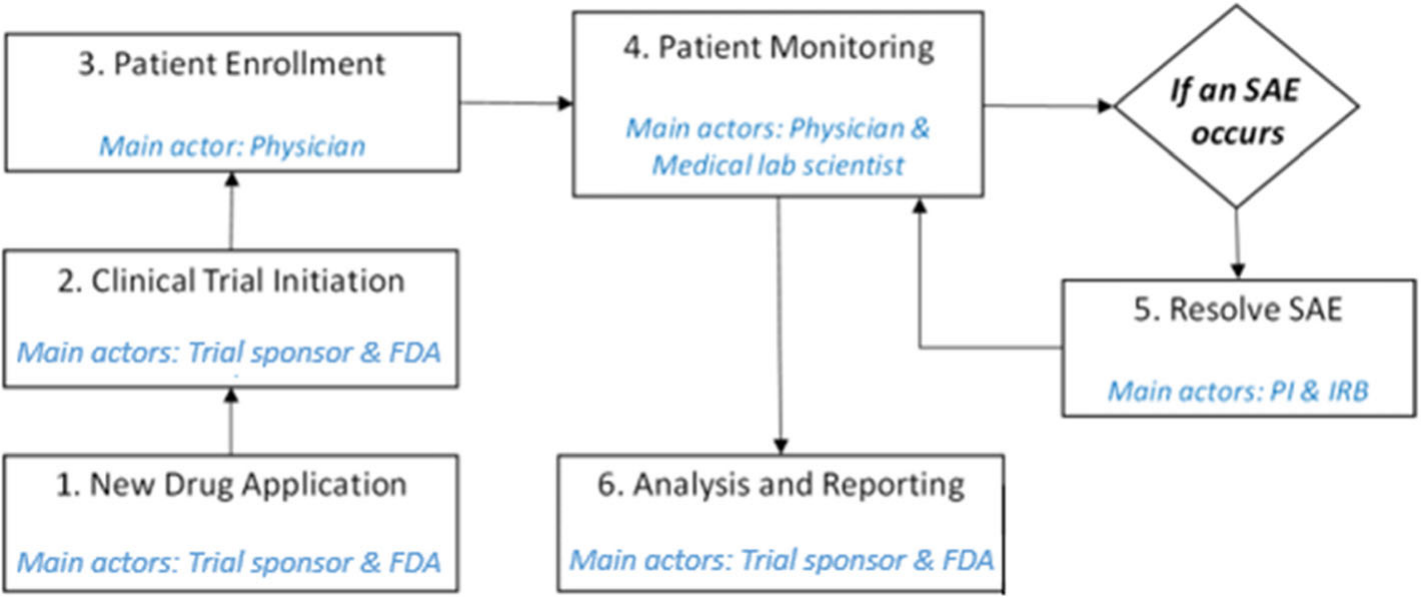
CT work process flow

Herein, we discuss the algorithms developed for each stage to capture the working principles of our proposed solution and accordingly develop the smart contract. Figure 5 highlights the main actors that would be able to interact with the contract for each stage respectively. This work process was designed under the assumption that the trial sponsor files an application to conduct only Phase I of the trial and hence, patient enrollment occurs only once. It could also be expanded to capture phase II and III of a CT process such that the contract could enable the enrollment stage to occur at the beginning of each new phase of the trial accordingly.

#### New drug application

Algorithm 1 describes the initial steps taken by the trial sponsor to initiate the CT process. This involves seeking approval upon submission of a new drug application. As can be seen, the trial sponsor is the only stakeholder allowed to file for the request, as the smart contract would reject all other unauthorized Ethereum addresses. Similarly, the decision can only be made by the FDA. Furthermore, it also lists the different set of information that is required to be fed into the blockchain as a transaction. This includes hashed files that link directly to IPFS.

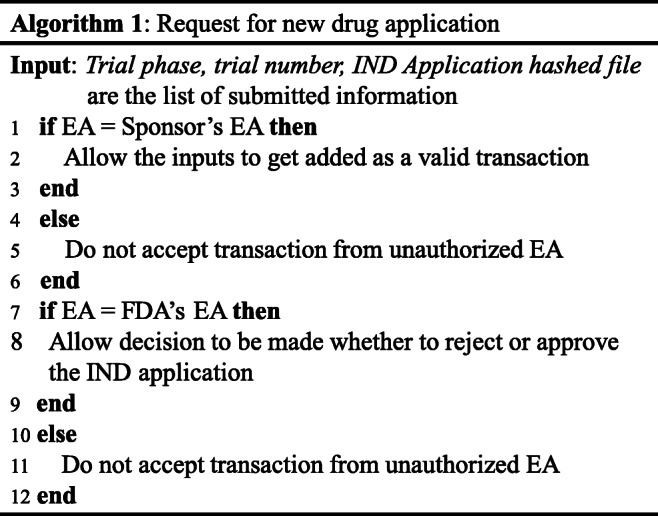



#### Clinical trial initiation

Algorithm 2 demonstrates that upon completion of a new drug application the trial sponsor can request for a CT initiation. It also demonstrates that the only stakeholders allowed to interact with the smart contract at this stage are the trial sponsor and FDA only. Also, the trial sponsor would be required to specify the information, such as the minimum number of patients required in the trial, protocol, and SOP.

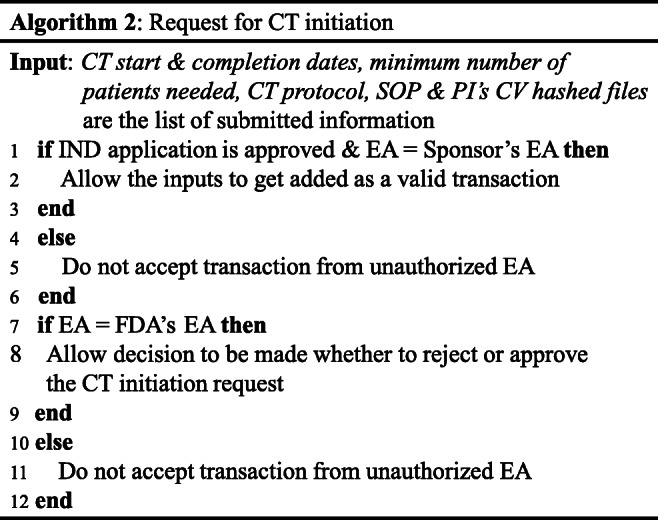



#### Patient enrollment

Algorithm 3 demonstrates that the patient enrollment process occurs only when the CT initiation request is approved. This algorithm lists the steps that would be taken during the enrollment stage of the CT. Firstly, only the assigned physician would be allowed to enrol the patients according to the inclusion criteria. The physician maps each patient’s address to respective details, such as medical history and informed consent. Then all the members in the network are notified whenever a new patient is registered into the trial. This stage is completed only when the number of patients enrolled meets or exceeds the cutoff number that was earlier stated in the CT initiation stage.

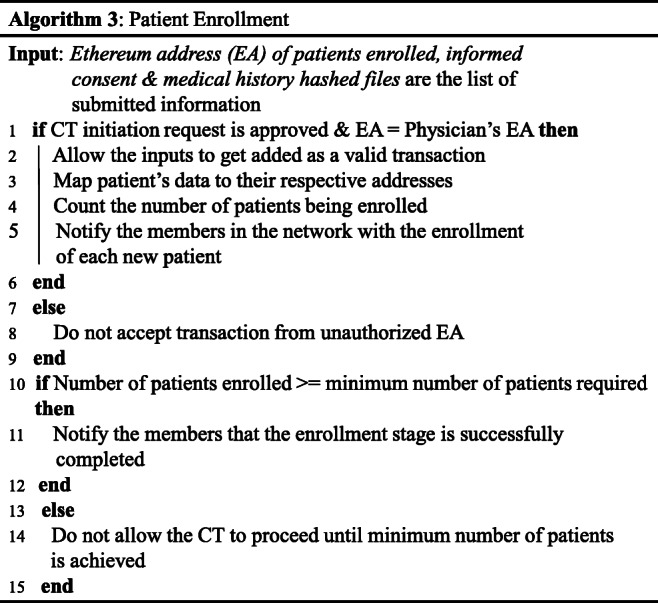



#### Patient monitoring

Algorithm 4 explains the monitoring process under normal circumstances upon the completion of the enrollment stage. The only stakeholders granted permissions are the physician and medical lab scientist to enable uploading the patient’s lab results, follow-up information and CRF upon each visit. This would help the physician in keeping track of their progress. Furthermore, if a patient were to drop or withdraw their informed consent during the trial then all stakeholders in the network would be notified and further monitoring of this patient would not be permissible. Thus, the data of the dropped patient would be available up to the point of when he or she was an active participant in the trial.

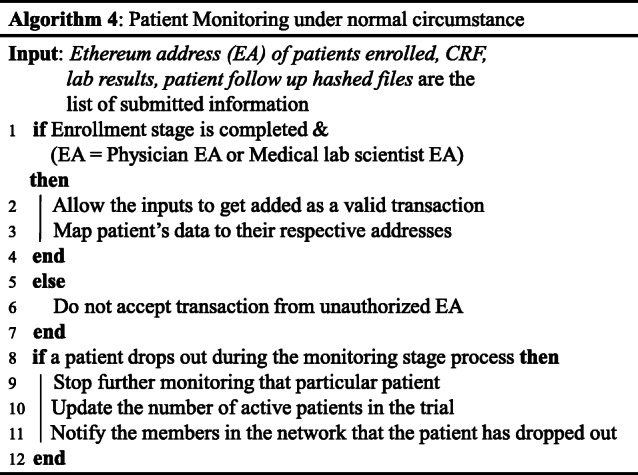



#### SAE occurrence

Algorithm 5 presents the steps that would be taken under the situation in which an SAE occurs during the trial conduction. The PI would upload the SAE reporting as per the meetings and investigations that would be carried between the physician and medical staff. Accordingly, the IRB would then decide whether to allow the trial to continue or stop the trial to ensure the safety of the participating patients.

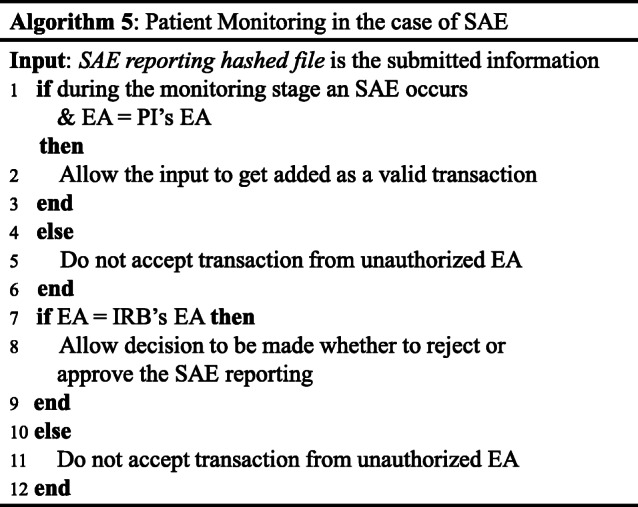



#### Analysis and reporting

Algorithm 6 shows that after the monitoring stage is completed and results analyzed, the trial sponsor submits a closure report to the FDA. The FDA then decides to either approve the newly tested drug for further investigation or discontinue the study.

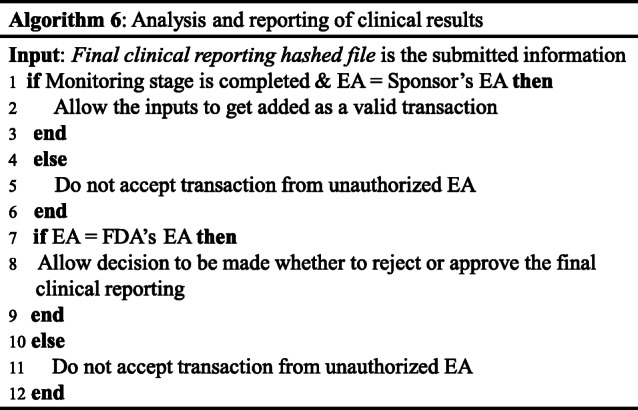



The above algorithms were then transformed into Ethereum smart contract using a browser-based compiler Remix IDE. The getter functions are used to retrieve information while setters are used to write data. Modifiers restrict the ability of certain members in the network to interact with a function. Events act as triggered alerts or signals to notify all members that a certain action has been taken. It should be noted that each member of the network is assigned a specific EA. Table 1 describes the functions used in the CT contract. The code of developed smart contract can be found on GitHub.^1^

**Table 1:** Description of functions used in the smart contract

CT Stage	Function	Input	Output	Permissions	Description
New Drug Application	**INDRequestedBySponsor**	Trial phase, trial number, IND Application hashed file	–	Trial Sponsor	File a new drug application request
	**SetINDApproval**	True/False	Alert	FDA	Approves or rejects the request based on the information provided
Clinical Trial Initiation	**ClinicalInitiationBySponsor**	CT start & completion dates, minimum number of patients needed, CT protocol, SOP & PI’s CV hashed files	–	Trial Sponsor	Request CT initiation
	**SetClinicalInitiaionDecision**	True/False	Alert	FDA	Approve or reject the request based on the information provided
Patient Enrollment	**PatientEnrollment**	EA of patients enrolled, informed consent & medical history hashed files	–	Physician	Enrol patients that meet the inclusion/exclusion criteria into the trial
	**GetPatientInfo**	–	Patients EAs	All participants in the network	Returns the address of patients enrolled in the trial
	**GetPatientsDetails**	Patient EA	Age, Informed consent & medical history hashed files		Returns the enrollment details about a particular patient address
	**CountPatients**	–	Number of patients enrolled		Returns the total number of patients participating in the trial
	**EnrollmentStageCompleted**	True/False	Alert	Physician	Notifies the participants on the completion of the enrollment stage
Patient Monitoring	**PatientMonitoring**	EA of patients enrolled, CRF, lab results, patient follow-up hashed files	–	Physician, Medical lab scientist	Update the details of patients with each visit
	**GetPatientMonitoringDetailPer Visit**	Patient EA, visit number	CRF, lab results, patient follow-up hashed files	All participants in the network	Returns the details of a patient based on a particular visit
	**dropPatient**	Patient EA	Alert	Physician	Drops the patient from the trial if he/she has discontinued the treatment
SAE Occurrence	**SetSAEStage**	SAE reporting hashed file	–	PI	Reports an occurrence of an SAE event
	**SAEApproval**	True/False	Alert	IRB	Approves or rejects the request based on the information provided
Analysis And Reporting	**ReportAnalysisStage**	Final clinical reporting hashed file	–	Trial Sponsor	Uploads the final CT findings
	**SetCTDecision**	True/False	Alert	FDA	Approves or rejects the request based on the information provided

## Results

In this section, we explain various test scenarios obtained upon the successful compilation of the smart contract. The CT process was tested in stages to ensure the process runs in smooth and sequential order.

### New drug application

**Fig. 6:**
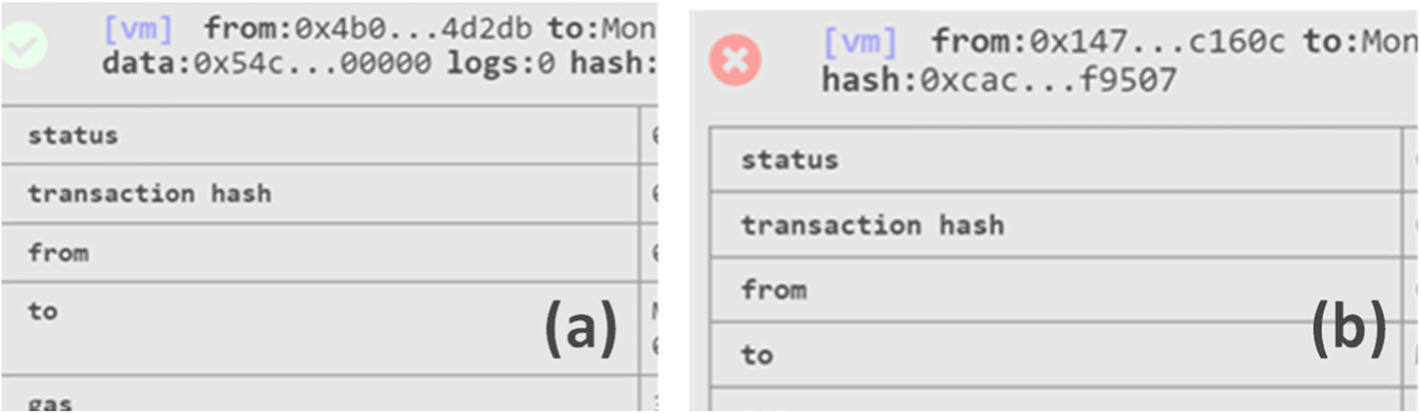
Testing modifiers wherein (**a**) function executed with no error as a trial sponsor was the assigned actor while in (**b**) error appears when an intended actor is not the FDA

At this initial stage, the modifiers were tested to ensure only the assigned actors are permitted to interact with a specific function. It can be seen in Fig. 6a that when the function *INDRequestedBySponsor()* was executed, no error was generated where a green tick mark indicated that the function was run by the trial sponsor as intended. However, in a case where another member in the network who is not given permission attempts to execute a function then an error is generated. For instance, when the function *SetINDApproval()* is not executed by the FDA then a red cross mark appears representing that the action cannot be completed as shown in Fig. 6b. This verifies that the restrictions imposed on each stakeholder at any stage in our proposed framework work as expected. Thus, only they would be allowed to add data into the network.

**Fig. 7:**
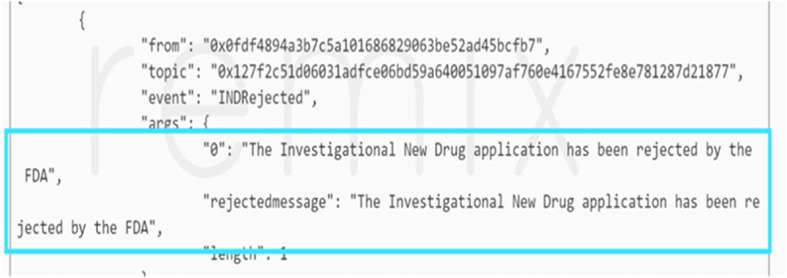
An event is triggered when the FDA makes a decision on the new drug application request

Secondly, the functionalities of events were tested to see whether they are triggered when certain actions are completed, such as the ending of each CT stage. Figure 7 shows that an event is prompted when the FDA makes a decision on the IND application where in this case the FDA rejected the application request.

### Clinical trial initiation

**Fig. 8:**
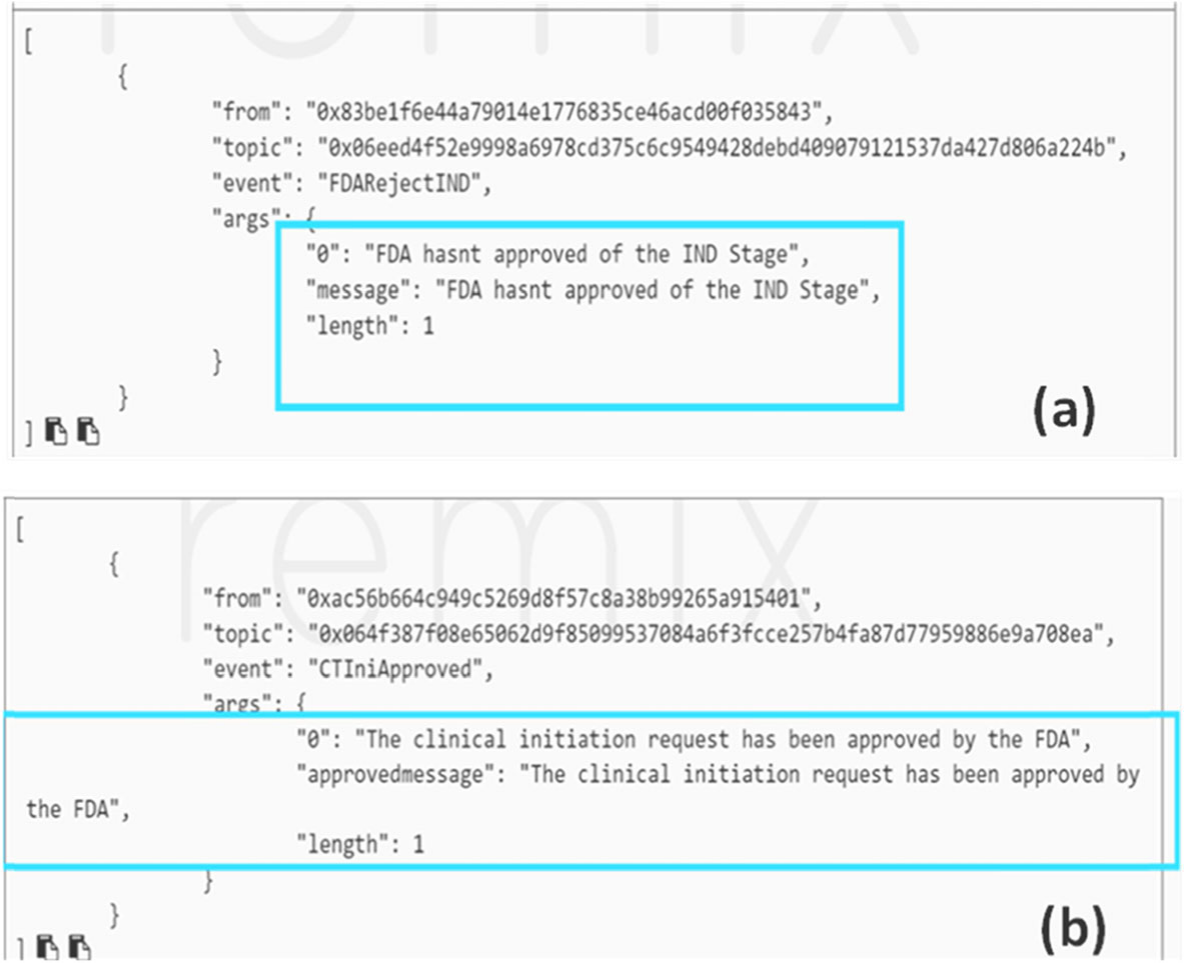
CT initiation stage cannot occur without the approval of the IND stage shown in (**a**) while in (**b**) its successful

The CT stages in the framework are expected to run in sequential order. Hence, Fig. 8 proves that the CT initiation stage does not commence until the IND application stage is completed and approved by the FDA.

### Patient enrollment

**Fig. 9:**
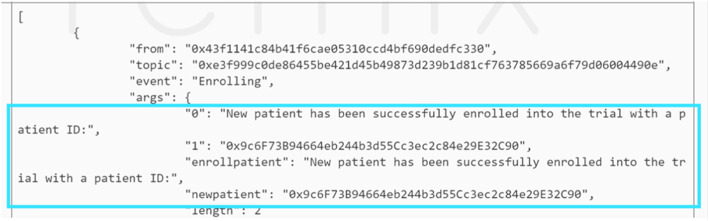
Enrollment of a new patient triggers an event that notifies all members that a new EA is registered

Likewise, the enrollment stage would be executed only after the CT initiation stage has been approved. Similarly, the enrollment stage would get executed only after the CT initiation stage has been approved. Additionally, an event is instantiated whenever a new patient is enrolled in the trial to notify all the stakeholders in the network. Figure 9 shows this event where it also displays the new patient’s EA which acts as the patient ID.

**Fig. 10:**
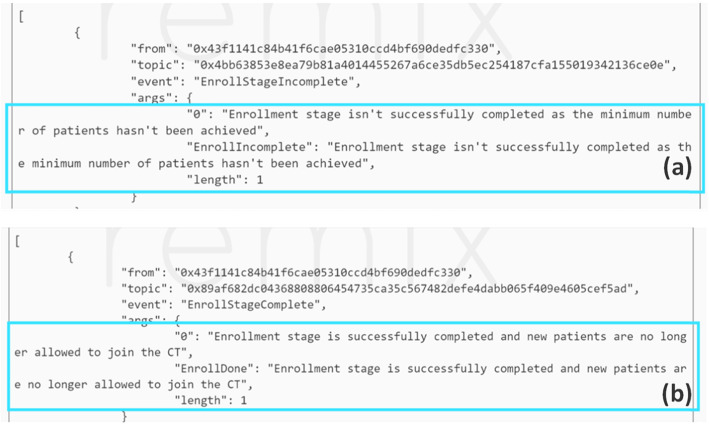
Event is triggered when the enrollment stage is completed wherein (**a**) it is not successful as the minimum number of patients is not met unlike in (**b**)

The completion of the enrollment stage requires that the number of patients enrolled meets or exceeds the minimum number stated in the CT initiation stage. This was tested by enabling the trial sponsor EA to specify five as the minimum number required in the initiation stage. However, in the enrollment stage, we made the physician EA enrol only four patients and proceed. Figure 10a shows that the event notifies all members in the network that this stage is incomplete. However, when the physician enrols one more patient then attempts to proceed to the next stage, an event is triggered allowing all stakeholders to know that the enrollment stage is completed as shown in Fig. 10b.

### Patient monitoring

**Fig. 11:**
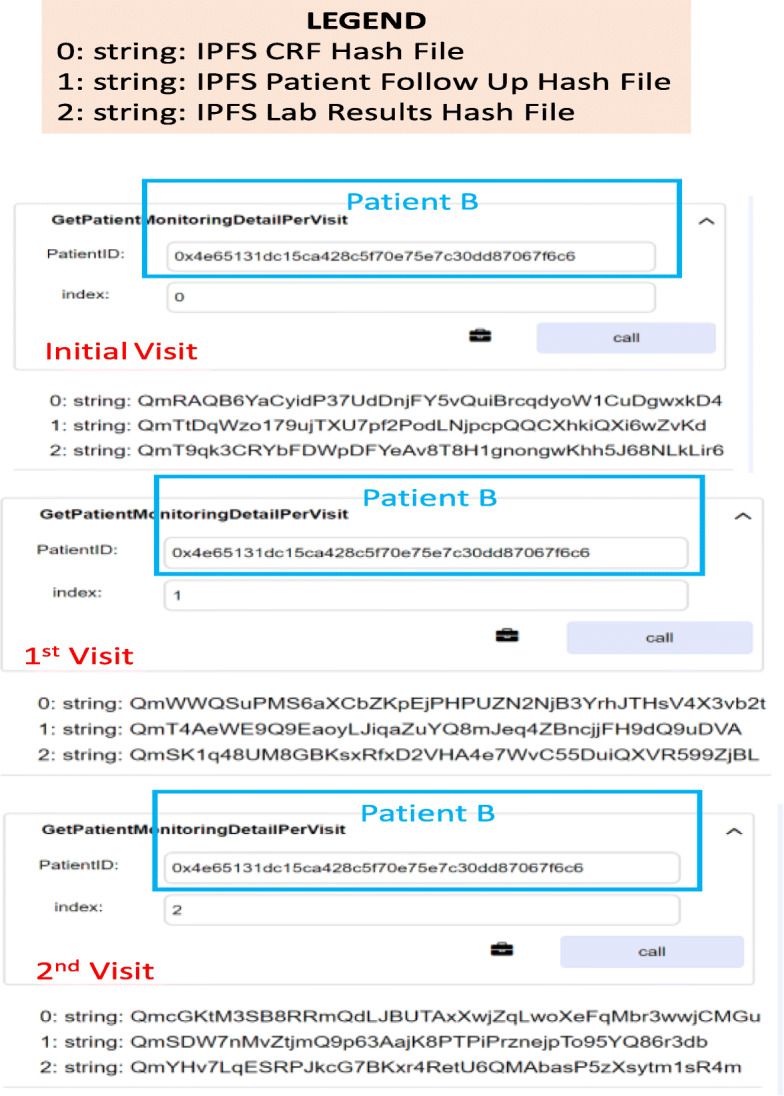
Retrieving the CT monitoring details of Patient B

This stage was tested by enabling the physician to upload Patient B’s vitals for each visit in the “IPFS Patient Follow-up and Lab results” hashed documents made to be able to track the progress. Figure 11 shows that a patient’s details according to the visit number can be retrieved as the details have been stored as an array of data type struct.

**Fig. 12:**
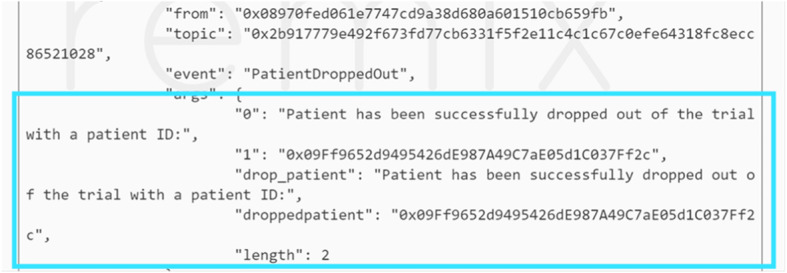
Event is triggered when an existing patient drops out of the CT

Moreover, this stage was tested in the case where a patient drops out of the trial. In such a case then the physician interacts with the smart contract and an event is triggered notifying the members in the network that Patient A with a particular EA has been dropped from the trial as shown in Fig. 12.

**Fig. 13:**
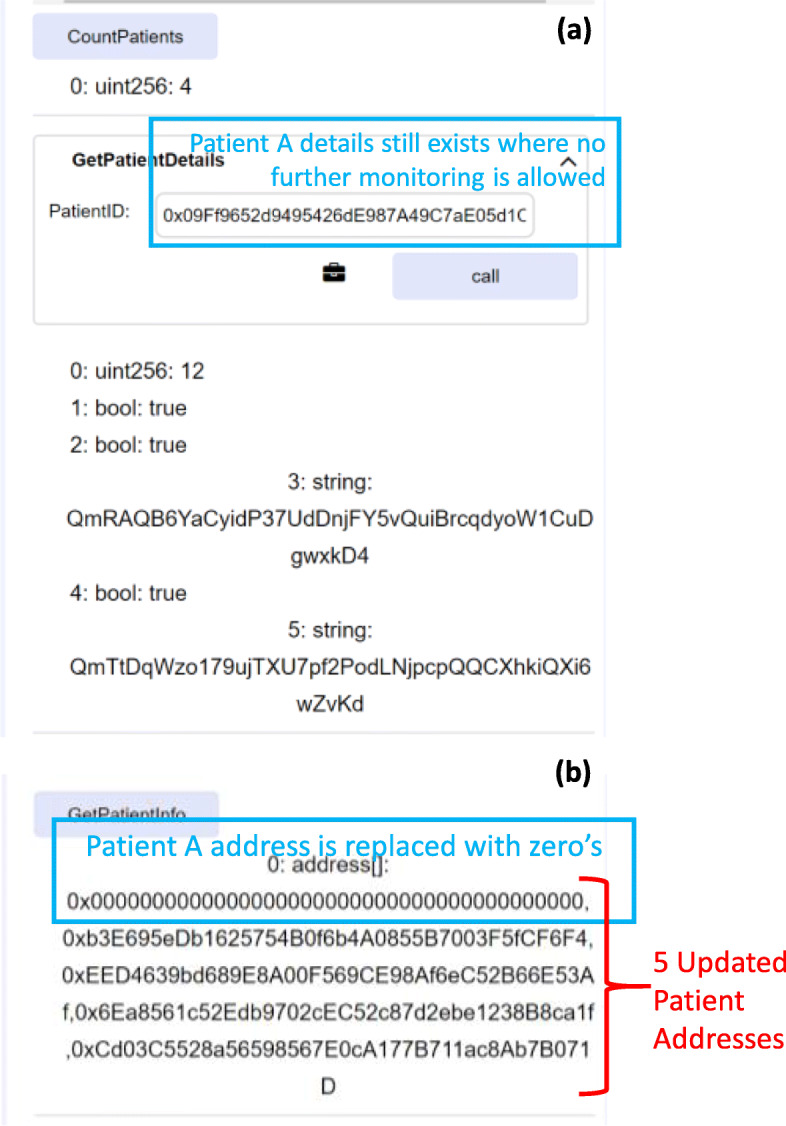
Retrieving (**a**) Patient A details after drop out (**b**) updated list of patient EAs

Furthermore, it was tested in the scenario where Patient A’s EA is inserted in the *GetPatientDetails()* getter function after it gets deleted. It was observed that a patient’s trial details are stored up to the point where the patient drops out as shown in Fig. 13a. Also, the CountPatients() getter function shows the updated number of patients who are currently participating in the trial where in this case the updated number is four. However, the getter function GetPatientInfo(), lists the patient addresses that are participants in the trial. Figure 13b shows the updated list of patient addresses where it is seen that Patient A’s address has been replaced by zero’s indicating that this patient is no longer an active participant in the trial.

**Fig. 14:**
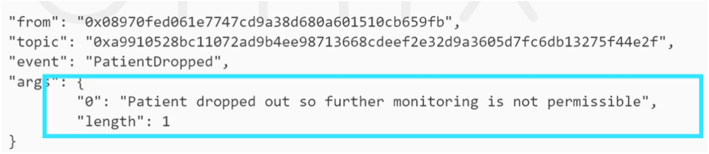
An event shows that a patient can no longer be monitored

A patient can no longer be monitored after having been removed from the trial and this was tested as the physician tried to monitor Patient A by uploading follow-up data. However, an event shown in Fig. 14 was triggered to notify the members that an attempt to further monitor this patient is against the protocol and hence, not permissible.

### SAE occurrence

**Fig. 15:**
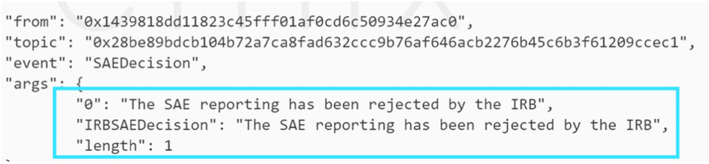
An event shows that the IRB rejected SAE report

The smart contract code was tested in the case where the SAE occurred, and the PI was reported by interacting with the smart contract. Thus, only the IRB has the right to decide regarding SAE reporting. The decision is announced, thereby triggering an event, as shown in Fig. 15, to alert the members on the status of the SAE where in this case, we tested the scenario in which the IRB rejects it.

### Analysis and reporting

**Fig. 16:**
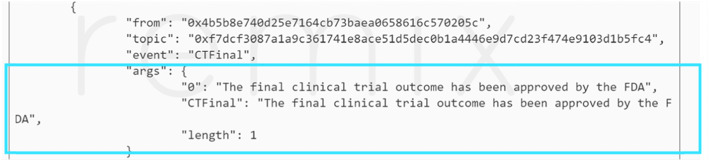
An event that shows the CT has been approved by FDA

The code was tested in the case where the “Patient Monitoring” stage has been completed successfully. So, the trial sponsor uploads the final CT report and the FDA makes a decision based on the trial outcomes. Figure 16 shows the tested case where the FDA approves the final CT outcome which consecutively triggers an event informing everyone that the trial has been approved.

## Discussion

The intrinsic properties of blockchain, such as distributed ledger, decentralization, anonymity, and immutability enable it to be exploited in various use cases ranging from supply chain and logistics to healthcare. One viable use case in the healthcare industry is CT data management. This is because blockchain can address crucial challenges, such as data management, protocol compliance data integrity, and transparency. The traditional CT data management is centralized and operated by a third party organization, such as CRO which creates an environment where trust is questionable. Moreover, data collected through such a system is more likely to be subjected to data entry errors and manipulation without being detected. Possible data includes, but is not limited to, informed consent, site monitoring, data analysis, and regulatory compliance. Thus, blockchain would empower regulatory authorities, such as the FDA allowing it to track clinical sites to see whether they are abiding by the submitted protocol. Blockchain has the potential to minimize malicious activities, such as selective reporting that could be in the form of under-reporting inconsistencies between final reporting and planned outcomes or non-significant outcomes. Therefore, blockchain could reemphasize data integrity as all participants are in a distributed network reinforcing transparency which in turn would discourage the falsification of data. We evaluated our proposed solution against important performance criteria, such as data management, protocol compliance, data integrity, and transparency.

### Data management

Clinical data management (CDM) is a crucial element in the conduction of a successful clinical trial. Using efficient data management systems aid in reducing the time required in collecting, cleaning, managing, and verifying data that comply with CT protocol and regulatory standards. It also involves identifying any missing information needed to accelerate the CT management process. Thus, having high-quality data would provide sufficient data for analysis and statistical quantification. Currently, there are various softwares available in the market for managing complex and large trials [33]; however, they are centralized and rely on third-parties to manage data. Our proposed blockchain-based solution can lighten the burden on the processes involved in a CT data management system. Moreover, every action taken by a stakeholder in our framework remains transparent to all members involved in the network. In this way, stakeholders cannot deny their actions at a later stage. This is because every validated transaction made by a stakeholder is signed by that particular stakeholder using their digital signature, which is directly generated using their private key through hashing.

### Protocol compliance

Following guidelines set by regulatory authorities, such as the FDA is essential in getting the trial approved. Ensuring that these rules are abided by the trial sponsor is necessary to protect the wellbeing of the patients involved in the trial. In a traditional clinical trial, the FDA used to conduct random and sudden visits to the clinical sites where these trials are conducted. This process is time-consuming, labor-intensive, cumbersome, and expensive. Our smart code captures these guidelines as a set of rules programmed into the blockchain. The test results of our code reveal that it ensures all actors involved in the CT follow a certain sequential procedure in which no actor is granted permission to proceed with the next stage without completing the prior stage, as shown in Fig. 5. For instance, without the clinical protocol approved by the FDA, the code does not move to the enrollment and monitoring stages of the CT process. Another example captured in our code is that without patients’ informed consent, their records cannot be updated and tracked during the monitoring stage. This ensures that the patient’s wellbeing is protected while simultaneously abiding by the IRB’s rules and regulations. Also, our code is generic enough, which can be tailored to accommodate all the rules that would comply with the regulatory rules and ethics. Moreover, the design of our contract ensures that the trial abides by the new protocol in case of an amendment is made into the CT protocol. This is necessary for running an efficient trial as all stakeholders would be aware of the new changes made in the protocol that they must abide by as the trial sponsor would re-upload a new document of the protocol which could be viewed through the IPFS. It should be noted that the nature of blockchain technology does not allow deletion of transactions. This is beneficial as stakeholders would be able to track and trace the changes made in the protocol throughout the CT lifetime in the IPFS and blockchain technologies.

### Data integrity

Data integrity is essential in CTs. This means that CT systems must be actively ready to look out for any mistake or malicious attempts. Our blockchain-based solution ensures that such mistakes or malicious attempts do not occur since the recorded data is validated using consensus algorithms. Also, the proposed solution ensures immutability using a hashing mechanism in which all the blocks are linked to each other through hash pointers that make it tamper-proof. Blockchain maintains all transaction logs that enable CT organizations to easily identify and trace back the origin of errors along with the person responsible for entering the inaccurate information. Hence, stored CT data on blockchain is considered as highly trustworthy.

### Transparency

Lack of CT transparency is a key issue because it hinders patients from getting the full picture of a treatment’s benefits and risks. In our proposed blockchain-based solution, the ledger is distributed among all stakeholders in a decentralized manner that minimizes the probability of clinical sites to selectively report optimistic results. In this way, our proposed solution enables collaboration between different clinical sites and stakeholders as all transactions can be viewed in real-time.

Our proposed framework captures the stakeholders that would interact with the Ethereum smart contract at each stage in the CT process in sequential order. This is illustrated in Fig. 2 which is later translated into a sequence diagram shown in Fig. 4. The proposed algorithms were translated into a smart contract where the code was sliced into six stages and chained together using if conditions. The functions used at each stage are listed in Table 1. This code’s functionality was tested, validated, and verified using Remix IDE. The various testing scenarios revealed that the code works in a sequential order ensuring that one stage does not proceed unless the previous stage is completed successfully as intended. Also, it verifies that the contract allowed only the participants who were permitted to interact with a specific function. Furthermore, events acted as an alert whenever an activity occurred or was completed, such as the enrollment of a new patient, withdrawal of informed consent by a trial participant or the drop out of the CT. Although the proposed solution is tested on the Ethereum blockchain platform, it can be deployed in private blockchain networks, i.e., Hyperledger and Quorum, using their native smart contract technologies. Also, our smart contract code can be extended to monitor multiple trial sites with minimal modifications. Hence, we can say the proposed solution is generic and can be implemented on multiple trial sites or a permissioned or permissionless blockchain depending on the needs of CT organizations.

Our smart contract code typically addresses the needs of a single-arm trial in which a pool of patients is selected with a targeted medical condition and given an experimental therapy. Subsequently, patients’ responses are monitored and tracked over a specific time. The implemented smart contract code is generic, which can be applied to various types of CT designs. The following changes can be made to the proposed smart contract to satisfy the needs of different CT designs.

### Placebo-controlled trials

In a placebo-controlled trial, patients with a targeted disease are identified and randomized to two treatments, such as active treatment and placebo. Thus, the monitoring stage in our contract would have to be modified to track the progress of these two groups separately. The *PatientMonitoring()* function would be split into two parts, such as *ActivePatientMonitoring()* and *PlaceboPatientMonitoring()*. The patients enrolled in the *PatientEnrollment()* function would be randomly registered into the two monitoring functions using their Ethereum addresses. It should be noted that the patients’ personally identifiable information would be protected and secured as only their addresses would be visible to the stakeholders involved in the network. Subsequently, the *ReportAnalysisStage() function* would analyze the results obtained from both treatment groups.

### Crossover trials

In a cross over design, patients are randomized into a sequence of treatments that would be sequentially administered during the treatment periods to compare different treatments. For instance, to compare treatments A and B over two periods, patients can be randomly divided into two groups in which group 1 patients are treated in the sequence of A followed by B; whereas, group 2 is treated in the sequence of B to A. In such a case, our code would divide the monitoring function into two periods in which former group of patients would be monitored using the *Sequence1PatientMonitoring()* function; whereas, the latter group would be monitored through *Sequence2PatientMonitoring()* function.

### Factorial trials

In such type of trials, researchers focus on analyzing the effect of interventions (alone or combined). For instance, researchers might be interested in studying interventions of A only, B only, both A and B, and either A or B. In these cases, we would have a function designated for monitoring each intervention accordingly. Therefore, the revised monitoring functions in our smart contract code would be as follows: *Intervention1PatientMonitoring()*, *Intervention2PatientMonitoring()*, *Intervention3PatientMonitoring()*, and *Intervention4PatientMonitoring(),* respectively.

### Noninferiority trials

This trial design is similar to the placebo-controlled trial where the treatment groups are divided into two categories; namely, active treatment and control. Unlike the placebo-controlled trial, the control group in this trial is not a placebo but an existing effective therapy, therefore, it is named as an active control group. Similarly, these trials are called active-controlled trials. For such type of trials, code would have an *ActivePatientMonitoring()* function and an *ActiveControlledPatientMonitoring()* function.

## Conclusions

In this paper, a blockchain solution using smart contracts has been proposed for managing data and process workflow in CTs. It provides a working proof-of-concept solution using smart contracts to address the data management, protocol compliance, transparency, anddata integrity challenges in a CT. This is because smart contracts operate autonomously without the intention of third parties. Also, it acts as a smart software agent making sure all decision points are fulfilled as intended. Furthermore, the proposed solution uses IPFS technology as a decentralized storage mechanism to store CT documents, such as protocol, informed consent, patient’s medical history etc. without the need of storing them directly in the blockchain. Our implementation shows that our framework can be highly effective in ensuring that the trial abides by the protocol and the functions are executed only by the stakeholders who are given permission. It also assures data integrity and promotes transparency and traceability of information among stakeholders. We also discussed how our code can be tailored to meet the needs of multi-site trials and various types of trial designs, such as placebo-controlled trials, crossover trials, factorial trials, and noninferiority trials. For future research, the proposed solution can be extended to incorporate Decentralized Applications (DApps) into the framework to aid in streamlining the interactions among different participants in the CT ecosystem. Moreover, our future research aims to deal with scalability issues and finding the exact cost of running a blockchain system in CTs research.

## Data Availability

Our smart contract code is made publicly available on Github: https://github.com/Clinical-Trial-Management/Clinical-Trial-Data-Management-via-Ethereum-Smart-Contract

## Abbreviations

CRFs: Case Report Forms
CTs: Clinical Trials
CRO: Contract Research Organization
DApps: Decentralized Applications
EA: Ethereum Address
FDA: Food and Drug Administration
IND: Investigational New Drug
IPFS: Interplanetary File System
IRB: Institutional Review Board
PI: Principal Investigator
SAE: Serious Adverse Event
SOP: Standard Operating Procedure (SOP)

## Footnotes

1 https://github.com/Clinical-Trial-Management/Clinical-Trial-Data-Management-via-Ethereum-Smart-Contract
